# Utility of a Novel Micro-Spraying Device for Intranasal Administration of Drug Solutions to Mice

**DOI:** 10.3390/pharmaceutics15112553

**Published:** 2023-10-29

**Authors:** Naoto Suzuki, Hiroaki Tanigawa, Taiki Nagatomo, Hiroko Miyagishi, Takanori Kanazawa, Toyofumi Suzuki, Yasuhiro Kosuge

**Affiliations:** 1 Laboratory of Pharmaceutics, School of Pharmacy, Nihon University, 7-7-1 Narashinodai, Funabashi 274-8555, Chiba, Japan; phhi20001@g.nihon-u.ac.jp (H.T.); nagatomo.taiki@nihon-u.ac.jp (T.N.); suzuki.toyofumi@nihon-u.ac.jp (T.S.); 2 Laboratory of Pharmacology, School of Pharmacy, Nihon University, 7-7-1 Narashinodai, Funabashi 274-8555, Chiba, Japan; miyagishi.hiroko@nihon-u.ac.jp; 3 Department of Clinical Pharmacology, Graduate School of Biomedical Sciences, Tokushima University, 1-78-1 Shoumachi, Tokushima 770-8505, Tokushima, Japan; kanazawa@tokushima-u.ac.jp

**Keywords:** intranasal administration, micro-spraying device, nose-to-brain, mice, inulin

## Abstract

Intranasal administration has attracted attention as a means of delivering drugs because it bypasses the blood–brain barrier. However, conventional intranasal administration of drug solutions to mice using the micropipette method (MP method) is complicated and time-consuming because it requires small doses to be administered under inhalation anesthesia. This study evaluated the effectiveness of a novel intranasal administration method using Micro FPS™, a novel micro-spraying device (the MSD method). The MSD method allowed more reliable administration of the solution to the nasal mucosa than the MP method did. The transfer of inulin, a model water-soluble macromolecule compound, to the olfactory bulb and brain (cerebrum, cerebellum, brainstem, and striatum) was similar with the two methods. It also allowed the drug to be administered in a shorter time. These results suggest that the MSD method is simpler and more rapid than the MP method for intranasal administration of drugs to mice and achieves comparable delivery of inulin to the olfactory bulb and brain. Therefore, the Micro FPS™ device is a potentially useful tool for intranasal drug administration to rodents and could facilitate the development of intranasal formulations, contributing to drug development for central nervous system diseases.

## 1. Introduction

### 1.1. Background

Intranasal administration of drugs through the nostrils to the nasal mucosa enables drug delivery to the systemic circulation via capillaries distributed locally in the nasal cavity and mucosa [1]. The nasal surface to which the drug adheres is covered with a thin mucosal layer with fewer proteolytic enzymes than the oral mucosa [2]. In addition, intranasal administration avoids hepatic first-pass effects. One of the most notable features of intranasal administration is the direct nose-to-brain (N2B) pathway, which enables efficient brain delivery of drugs with a significantly limited blood-to-brain transfer, bypassing the blood–brain barrier [3,4,5,6]. These features enable the delivery of novel modalities, such as peptides and nucleic acid drugs, to the central nervous system [7,8] and suppress systemic side effects of drugs that occur when administered via conventional administration methods [6,9]. Therefore, intranasal administration presents the potential for further development of formulations targeting the central nervous system, local nasal cavity, and systemic circulation.

In drug development, pharmacological evaluation of new drugs is generally conducted in rodent species such as mice and rats. However, standardized administration conditions (e.g., volume, time, and administration rate) for intranasal administration to rodents have not yet been established, and administration methods differ among researchers [10]. The lack of standardized administration methods is a cause for concern because differences in administration methods among researchers may have a considerable effect on drug retention in the nasal mucosa and drug delivery to the brain due to the complexity of the nasal cavity [11]. The most common method of intranasal administration to rodents is the micropipette (MP) method. In this method, 10–20 µL of drug solution is administered into the nasal cavity of mice, which has a volume of approximately 30 µL, under inhalation anesthesia with isoflurane [12] using a micropipette 10–20 times into alternating nostrils, and the drug is absorbed into the nasal mucosa without an excess amount of solution being excreted into the pharynx.

### 1.2. Rationale

Previously, we developed an openable inhalation mask that allows intranasal administration even under inhalation anesthesia. This method enabled the administration of drug solutions in the same position to rodents under inhalation anesthesia. Moreover, this minimized the leakage of drug solutions from the nasal cavity to the pharynx and variability due to the technique [10,13]. Fukuda et al. [14] evaluated the brain translocation of inulin, which is a model water-soluble macromolecule, using these MP-technology-based dosing methods. Kurano et al. [15,16] demonstrated the migration of liposomes with different surface charges intranasally administered via the MP method to the brain and spinal cord and the therapeutic effect of intranasal administration of N-acetyl-L-cysteine, an antioxidant loaded on a polymeric nanocarrier, in a mouse model of amyotrophic sclerosis. The MP method is widely used to evaluate the kinetics or therapeutic effects of intranasally administered formulations based on their physical properties. However, these MP methods are complicated because the maximum volume that can be administered per dose is minimal (1–2 μL) and is administered into the right and left nostrils at 1 min intervals, implying that 10–20 min are required to administer the entire solution. Moreover, as there is always a time lag between the start and end of administration, the amount of drug distributed on the nasal surface and its residence time may differ from those of actual intranasal administration, which may affect pharmacokinetics and drug effects. To resolve these issues, Ullah et al. [17] investigated intranasal administration under awake conditions. Intranasal administration under awake conditions can be performed in a shorter time because it eliminates the need for anesthesia and other time-consuming procedures [17,18]. However, the results depend on the specific equipment used and the technical proficiency of the operator. Therefore, this method cannot be universally applied. Additionally, mice require time to acclimatize before intranasal administration under awake conditions. Notably, the distribution of the administered drug is highly variable; therefore, awake administration is not a satisfactory method.

### 1.3. Objectives

**Figure 1:**
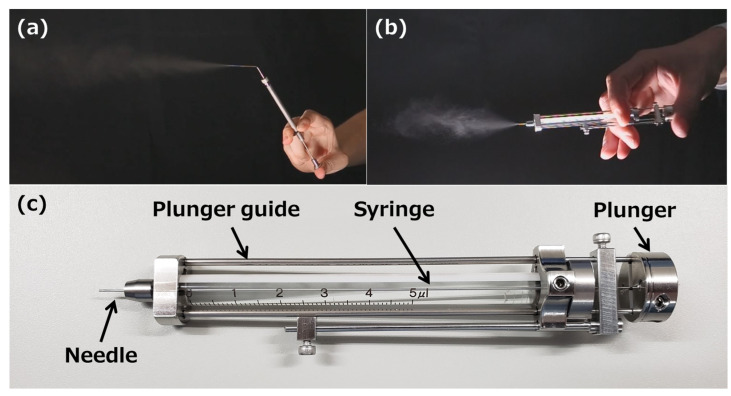
Spraying demonstration of (**a**) fine particle sprayer and (**b**) Micro FPS™. (**c**) Structure of Micro FPS™.

Micro FPS™, a new micro-spraying device (MSD; Figure 1) for intranasal administration of drugs to mice [19], is capable of spraying drug solutions in the range of 1–5 µL in 1 µL increments. This is otherwise difficult to achieve with the fine particle sprayers used for transpulmonary administration to rodents. This new MSD is expected to reduce differences in intranasal administration techniques among experimenters and simplify intranasal administration. Therefore, to clarify the usefulness of Micro FPS™ as a new intranasal administration device, we compared the time required for intranasal administration, nature of the distribution of the drug solution in the nasal cavity of mice, and transfer of inulin, a model water-soluble macromolecule compound, into the olfactory bulb or brain (cerebrum, cerebellum, brainstem, and striatum) to those of the MP method.

In this study, we investigated the intranasal distribution of drug solution in the nasal cavity of mice and brain migration of inulin, a model water-soluble middle molecule, administered intranasally using Micro FPS™ MSD to evaluate the usefulness of this MSD for intranasal delivery of drug solutions to mice.

## 2. Materials and Methods

### 2.1. Reagents and Materials

A solution of 0.4% trypan blue and radiolabeled inulin ([Carboxyl-14C]-inulin, molecular weight approximately 5000, 2 mCi/g; purity > 99%, Muromachi Kikai Co. Ltd., Tokyo, Japan) added to the drug solution was purchased from Thermo Fisher Scientific Corporation (Tokyo, Japan) and American Radiolabeled Chemicals (St Louis, MO, USA), respectively. The Micro FPS™ (MFPS-01-A1, MSD) microdose atomizer (Toray Precision, Inc., Shiga, Japan) was used for intranasal administration of the solution to mice. Table 1 lists the spray characteristics of Micro FPS™.

### 2.2. Animals

All animal experiments were conducted under the guidelines approved by the Nihon University Animal Care and Use Committee (Tokyo, Japan, experiment number #AP22PHA013-1). Seven-week-old male mice (Deutschland, Denken, and Yoken strains) were obtained from Japan SLC, Inc. (Shizuoka, Japan). Mice were housed under a light–dark cycle of 25 ± 1 °C, 55 ± 10% humidity, and 12 h of illumination (8:00–20:00), with ad libitum access to feed and water. Male mice weighing 35–45 g were used in the subsequent studies.

### 2.3. Intranasal Distribution of Trypan Blue Solution

**Figure 2:**
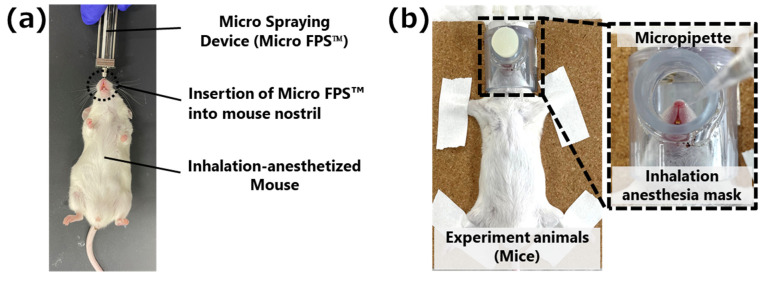
Comparison of intranasal administration methodologies and dosing schedule of each method. (**a**) Micro-spraying device (MSD) method: 1–5 µL administered into each nostril at a time using Micro FPS^TM^. (**b**) Micropipette (MP) method: 1–2 µL administered into each nostril at 1-min intervals via micropipette.

In the MSD method, the needle of the MSD was inserted into the right nostril of a mouse immobilized in the supine position under inhalation anesthesia with isoflurane using an openable inhalation mask (SN-487-70-09, Shinano Seisakusyo, Tokyo, Japan) [10] and 4 µL of 0.4% trypan blue, an azo dye similar to that used for nasal staining in the previous report [20], and the solution was sprayed into the nasal cavity of the mouse (Figure 2a). Mice were removed from the anesthesia mask during administration. In the MP method as a reference method, droplets of 0.4% trypan blue solution were formed at the tip of a micropipette and aspirated by spontaneous breathing of the anesthetized mice [10] (Figure 2b). After opening the silicon cap of the mask, 1 µL of 0.4% trypan blue solution was administered into the right nostril of the mouse at 1-min intervals from the opening of the inhalation mask to make a total volume of 4 µL. After the administration of each method, the head was cut in a sagittal plane, and the distribution of trypan blue adhering to the inside of the nasal cavity was captured. This image was analyzed using ImageJ software downloaded from the NIH website [21], and the adhesion rate of the trypan blue solution to the entire nasal cavity was calculated using Equation (1):

(1) $$\mathrm{Adhesion}\;\mathrm{ratio}\;\mathrm{of}\;\mathrm{trypan}\;\mathrm{blue}\;\mathrm{solution}\;\mathrm{in}\;\mathrm{the}\;\mathrm{mice}\;\mathrm{nasal}\;\mathrm{cavity}\;\left(\%\right)\;=\frac{\mathrm{Trypan}\;\mathrm{blue}\;\mathrm{adhered}\;\mathrm{area}}{\mathrm{Nasal}\;\mathrm{cavity}\;\mathrm{area}}$$

### 2.4. Measurement of the Amount of [14C]-Inulin to the Brain and Olfactory Bulb after Intranasal Administration

A total of 10 µL of phosphate-buffered saline solution containing 5 µCi/mL (400 µM) of [14C]-inulin was administered intranasally to the mice using the MP or MSD method. In the MSD method, Micro FPS™ was filled with 5 µL of the drug solution and administered intranasally into the right and left nasal cavities once each. In the MP method, 1.25 µL of drug solution was administered eight times into the right and left nasal cavities at 1-min intervals, based on the methods used in previous studies [10,14]. Thirty minutes after each administration, isolated brain tissue was divided into two parts: the olfactory bulb and the rest of the brain, including the cerebrum, cerebellum, brainstem, and striatum. Each sample was dissolved in Solusol™ (National Diagnostics, GA, USA), a tissue solubilizer, at 55 °C for 2 h. Then, 10 mL of Hionic-flour™ (Waltham, MA, USA) known as the liquid scintillation cocktail was added to the samples. The drug solution equivalent to the dosage was dissolved in 3 mL of Pico-flour™ 40 (Waltham, MA, USA). To determine the [14C]-inulin radioactivity in the olfactory bulb and brain, a liquid scintillation counter was used (Tri-Carb 4810TR, PerkinElmer, Shelton, CT, USA). All experiments using isotopes were conducted in accordance with the Isotope Center Use Plan, which was reviewed and approved by the Isotope Center of Nihon University, School of Pharmacy (experiment #23-01, 02).

### 2.5. Statistical Analysis

JMP Version 14 (SAS Institute Inc., Cary, NC, USA) was used for statistical analysis. The significance of the difference among three groups (untreated or treated using the MP or MSD method) in the adhesion ratio of trypan blue solution in the nasal cavity of the mice was determined using analysis of variance with Tukey’s test for multiple comparisons. *p* values < 0.05 were considered statistically significant. Additionally, the *t*-test for independent samples was used to evaluate the significance of the difference between the MP and MSD methods in the transfer of [14C]-inulin to the olfactory bulb and brain.

## 3. Results

### 3.1. Adhesion of 0.4% Trypan Blue Solution to the Nasal Cavity of Mice

**Figure 3:**
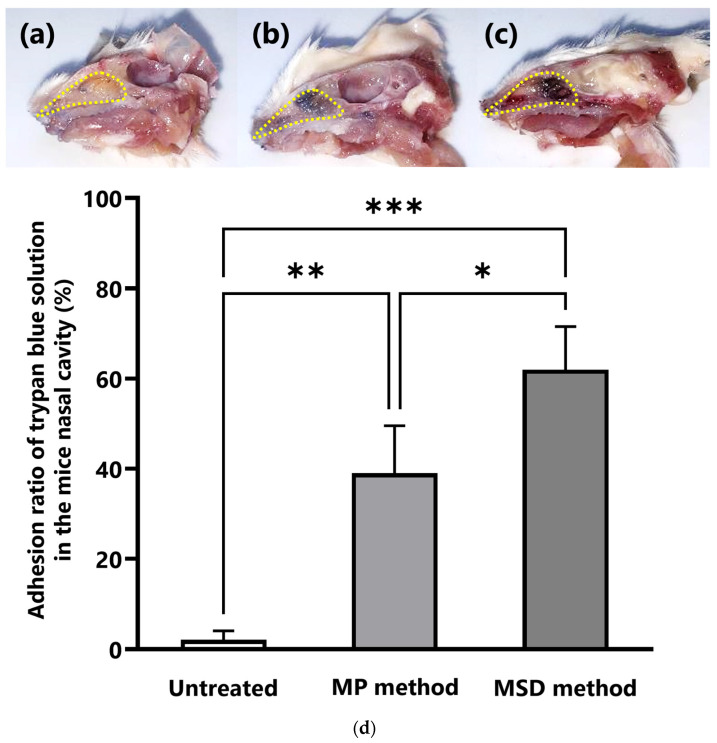
Distribution of the dosing solution in the nasal cavity of mice according to the intranasal administration methods. (**a**) Untreated; (**b**) MP method; (**c**) MSD method. (**d**) Adhesion ratio according to the intranasal administration methods. The distribution of trypan blue in the nasal cavity was confirmed from a sagittal section of the head of mice administered 0.4% trypan blue intranasally. In (**a**–**c**), the yellow dotted line indicates the nasal cavity of mice. The significance of the differences in the mean values of the three groups was estimated using analysis of variance with Tukey’s test. * *p* < 0.05, ** *p* < 0.01, *** *p* < 0.001. Each bar represents mean ± standard error of three treated mice. MP, micropipette; MSD, micro-spraying device.

In this experiment, the 0.4% trypan blue solution was administered intranasally to stain the nasal cavity, as reported by Kumar et al. [20], and the adhesion of the trypan blue solution to the nasal cavity was calculated. We confirmed that 0.4% trypan blue solution can be sprayed using Micro FPS™. Unlike that in the mice with no administered trypan blue solution (Figure 3a), the administered trypan blue was distributed between the nostrils and nasal cavity and in the anterior part of the nasal cavity using the MP method (Figure 3b). In contrast, the MSD method showed no distribution of trypan blue between the nostrils and nasal cavity, and most of the sprayed trypan blue solution reached deep within the nasal cavity, physically close to the brain tissue (Figure 3c). Compared with that using the MP method, the adhesion rate of trypan blue in the nasal cavity using the MSD method increased significantly by approximately 1.5 times (Figure 3d).

### 3.2. Transfer of Inulin from the Nasal Cavity to the Brain of Mice

**Figure 4:**
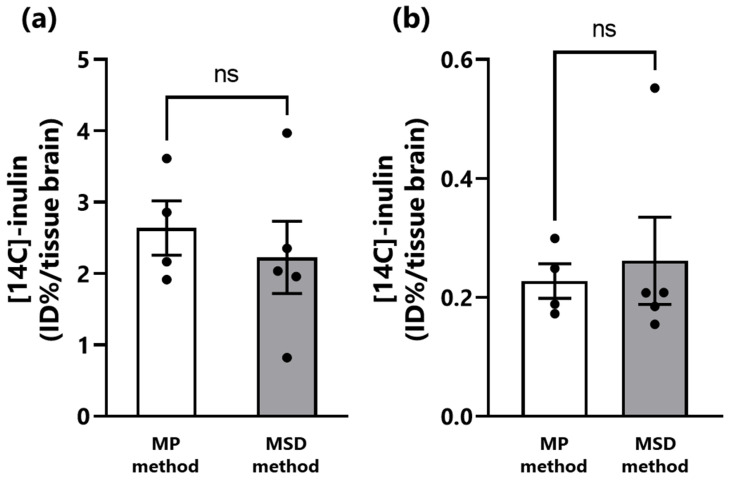
Comparison of the transfer of inulin to the olfactory bulb and brain of mice using the MP and MSD methods of intranasal administration. (**a**) The olfactory bulb; (**b**) the brain. The ratio of [14C]-inulin distributed to the olfactory bulb and brain relative to the total amount of [14C]-inulin administered by each method was divided by the tissue weight and expressed as %ID/g. Each bar represents the mean ± standard error of four mice treated using the micropipette (MP) method or five mice treated using the micro-spraying device (MSD) method. ns; not significant (*p* > 0.05).

Subsequently, we compared the brain transfer of inulin. This water-soluble macromolecule model compound has previously been shown to be transferred to the brain via the N2B route using the MP method of intranasal administration [14]. Micro FPS™ is also capable of spraying an inulin-containing drug solution. The results showed no significant difference between the MP and MSD methods in the amount of inulin transferred to the olfactory bulb and brain 30 min after intranasal administration, indicating that the method of administration did not affect the transfer of inulin to the brain (Figure 4).

Compared with that of the MP method, the MSD method significantly reduced the time required for administration (Table 2). In this experiment, the time required for intranasal administration of 10 µL of drug solution was approximately 60 s for the MSD method and approximately 190 s for the MP method. 

## 4. Discussion

Recently, intranasal administration has been attracting attention as an administration method that can deliver drugs directly to the central nervous system [22]. Notably, the blood–brain barrier, comprising brain capillary endothelial cells, pericytes, and astrocytes [23,24,25], restricts the delivery of drugs to the brain using conventional methods. The therapeutic effects of these drugs administered intranasally to rodents using the micropipette (MP) method and their translocation into the brain and blood have been reported [11,14,26,27,28]. The MP method is more widely used than other intranasal administration methods because it is simple and relatively easy. However, the time required for administration through the MP method and differences in the proficiency of the administration technique among the experimenters are barriers to research. Our research group has reported the development of masks for inhalation anesthesia and a reverse canulation method to shorten the administration time and minimize the influence of inter-experimenter skills [10]. However, these investigations only solve some of the problems associated with intranasal administration. In this study, we focused on Micro FPS™, a new micro-atomizer developed by Toray Precision Inc. We examined its applicability for intranasal administration of drugs to mice, which are commonly used as experimental animals.

First, we observed the intranasal distribution of the drug solution administered using each intranasal administration method. Similar to Evans blue, trypan blue, which is also an azo dye, could clearly stain the nasal cavity. The MSD method showed no distribution of trypan blue between the nostrils and nasal cavity, and most of the drug solution reached deep within the nasal cavity adjacent to the brain tissue. This is because the length of the Micro FPS™ needle is approximately 7 mm, which corresponds to the length between the nostrils and nasal cavity of a mouse, allowing the needle tip to be inserted into the nasal cavity and spray the drug solution. In contrast, with the MP method, the trypan blue was administered intranasally as the mice spontaneously breathed, and trypan blue remained in the nostril and the anterior part of the nasal cavity. Therefore, the adhesion rate of trypan blue solution in the nasal cavity of mice was significantly higher using the MSD method than using the MP method. The MSD method was able to administer drugs intensively deep into the nasal cavity, to the area where the olfactory nerve endings are distributed. The olfactory nerve pathway via the olfactory nerve from the olfactory region located in the deepest part of the nasal cavity and the trigeminal pathway via the trigeminal nerve distributed in the submucosa of the nasal cavity are important pathways in N2B drug transfer [11,29,30]. Additionally, differences in the volume of the rodent’s nasal cavity may affect drug delivery to the olfactory bulb [31]. The MSD method using Micro FPS™, which allows intensive spray of drug solution into the olfactory region, facilitates N2B transfer, without the distribution of drug solution between the nostril and nasal cavity, suggesting that drug solution can be efficiently administered to the olfactory area regardless of the nasal cavity volume. Flamm et al. [32] developed a catheter-based application that can deliver drugs precisely to the olfactory region. This device is beneficial from a pharmacokinetic or formulation perspective because it allows direct administration of drug solutions with various physical properties. However, drugs are administered to the nasal cavity as droplets using atomizers in most practical applications. It has been reported that the size of the sprayed droplet affects drug deposition in the nasal cavity [33,34]. Therefore, intranasal administration using Micro FPS™, which can atomize the drug solution as fine droplets, is a beneficial method for designing intranasal formulations. Additionally, when the drug is sprayed as droplets using Micro FPS™, fine particles may reach respiratory organs such as the airways, lungs and gastrointestinal tract when the mice inhale. Since A sprays droplets measuring several tens of micrometers, the risk of reaching the pharyngeal side or respiratory tract is considered low. However, along with the nasal cavity, the respiratory and gastrointestinal tracts also need to be evaluated in further studies for the possibility of secondary side effects owing to inhalation or swallowing of the drug.

The brain transfer of inulin to the olfactory bulb and brain after intranasal administration was compared between the two methods. Our group has previously reported that intranasal administration of 25 µL of inulin solution using the MP or reverse canulation method resulted in the transfer of 1.5–3.5% ID/g tissue to the olfactory bulb and 0.1–2.7% ID/g tissue to the brain and that the rate of inulin transfer to each tissue increased with decreasing volume of administration using the reverse canulation method [14]. In the current study, the degree of inulin brain transfer using the MP method was similar to that reported previously. The level of inulin transfer to the olfactory bulb and brain was similar using the MP and MSD methods. These results indicate that the MSD method is as effective as the MP method for the delivery of drug solution into the nasal cavity of mice. Despite differences in the nasal distribution of the drug solution due to the administration method, the brain transfer of inulin did not differ regardless of the method used. Intranasal administration is a useful method of administration to improve brain delivery of water-soluble low-molecular-weight drugs with low mucosal permeability [35]. However, the nasal mucosal permeability of drugs generally tends to decrease with increasing molecular weight [36,37,38]. In addition, we confirmed that the amount of intranasally administered inulin transferred to the olfactory bulb and brain was constant regardless of the dose of inulin. The inulin used in this study as a model drug is water-soluble and has a molecular weight of 5000 Da, because of which the efficiency of intranasal delivery to the brain was limited, and the difference in the distribution of the administered solution in the nasal cavity due to the intranasal administration method did not affect the transfer of inulin to the olfactory bulb or brain. Therefore, despite the differences in the distribution of the drug in the nasal cavity, no difference in the amount of inulin transferred from the nose to the olfactory bulb or brain was observed. Further research is required to examine the effects of the method of intranasal administration and nasal distribution of the drug solution on the transfer of low-molecular-weight or lipophilic compounds with a high contribution to brain delivery via the N2B route to the blood and various tissues, especially into various regions of the central brain [39,40]. Micro FPS™ has not yet been evaluated in rats. Rats have a larger nasal cavity volume than that of mice. Micro FPS™ can also be used in rats for intranasal administration of candidate compounds for new drugs. However, the needle length of Micro FPS™ may need to be changed because the length from the nostrils to the nasal cavity is longer in rats than in mice.

In addition, we compared the time required for intranasal administration of 10 µL of drug solution to mice. The MSD method reduced the time required for intranasal administration by 2 min, from 3 min to 1 min, compared with that of the MP method, in which 1.25 µL of the drug was administered into the right and left nasal cavities alternately, followed by a 1-min interval between administrations. This is because the maximum volume of Micro FPS™ is 5 µL per spray and only one spray in each nostril was needed to administer the total drug dose. Therefore, intranasal administration to rats, which have a larger nasal cavity volume than that of mice, using the MSD method is likely to shorten the administration time further. Micro FPS™ also allowed for one-handed syringe filling and spraying from the device. Ullah et al. [17] reported a novel method in which mice are immobilized in a particular device that maintains them in a condition suitable for brain delivery [41] of siRNA by intranasal administration. This administration method is efficient, allowing the substance to be administered intranasally to 4–8 mice at the same time. However, it requires a special device to immobilize the mice and a micropipette for intranasal administration; therefore, it requires approximately the same amount of time as the MP method. In contrast, Micro FPS™ allows 1–5 µL of drug solution to be sprayed intranasally at a time, thus reducing the administration time (Table 2). These advantages of increased speed and reduced costs are extremely important in terms of practical application. While the nasal retention property is essential for brain-targeted formulations by adding high-molecular-weight thickener or gelling agents, spraying viscous solutions with MSD has been difficult, so further improvement of the device is expected in the future. The development of an MSD that can spray a viscous solution is expected to accelerate the design of intranasal formulations for N2B administration. This could contribute to the development of drugs to treat central nervous system diseases in humans.

## 5. Conclusions

In this study, we investigated the usefulness of an intranasal administration method using Micro FPS™, a novel micro-atomizer, as an alternative to the MP method for intranasal administration of drug solutions to rodents, in this case using mice. Intranasal administration of drug solutions was more rapid and simpler using the MSD method than that using the MP method. Using the MSD method, all of the administered drug solution could be sprayed into the nasal cavity, and most of it was deposited in the olfactory area, which is involved in N2B transfer. Furthermore, there was no difference in the amount of inulin transferred to the brain after intranasal administration by each method, suggesting that Micro FPS™ can be used for intranasal administration of drug solutions to rodents in the same way as the conventional MP method. In conclusion, intranasal administration of drugs to mice using Micro FPS™ is a useful method to investigate drug delivery to the central nervous system via the N2B pathway. Further studies using water-soluble middle-molecule model compounds and drugs with various characteristics are expected to provide a useful tool for developing nasal formulations based on tests using rodents.

## Figures and Tables

**Table 1:** Specifications (spray characteristics, needle size, and device weight) of the novel micro-spraying device Micro FPS™ [19].

Spray Characteristics	
Minimum spray volume	1 μL
Maximum spray volume	5 μL
Average spray angle	43.8°
Average atomizing particle size	12.97 μm
Needle size and device weight	
Sprayable viscosity at 20 °C	1.000 mPa·s
Needle outer diameter	0.52 mm
Needle length	7.0 mm
Device weight	45.5 g

**Table 2:** Time for intranasal administration of drug solution by each method. S.E.M., standard error of the mean.

Time for Administration (s)	*n*								Mean	S.E.M.
	1	2	3	4	5	6	7	8		
MSD method	57	55	51	57	63	57	…	…	56.67	3.54
MP method	197	191	194	191	190	192	193	194	192.75	2.11

## Data Availability

Not applicable.

## Abbreviations

MP	micropipette
MSD	micro-spraying device
N2B	nose-to-brain
